# Effect of age on lung adaptation to high-altitude hypoxia in Tibetan sheep

**DOI:** 10.3389/fvets.2024.1458774

**Published:** 2024-12-09

**Authors:** Pengfei Zhao, Shaobin Li, Fangfang Zhao, Jiang Hu, Jiqing Wang, Xiu Liu, Zhidong Zhao, Mingna Li, Yuzhu Luo

**Affiliations:** ^1^Gansu Key Laboratory of Herbivorous Animal Biotechnology, Faculty of Animal Science and Technology, Gansu Agricultural University, Lanzhou, China; ^2^Faculty of Chemistry and Life Sciences, Gansu Minzu Normal University, Hezuo, China

**Keywords:** Tibetan sheep, high-altitude hypoxia, adaptation, lung, age

## Abstract

After prolonged adaptation to high-altitude environments, Tibetan sheep have developed a robust capacity to withstand hypobaric hypoxia. Compared to low-altitude sheep, various organs and tissues in Tibetan sheep have undergone significant adaptive remodeling, particularly in the lungs. However, whether lambs and adult Tibetan sheep exhibit similar adaptations to high-altitude hypoxia remains unclear. In this study, we selected six lambs (4 months old) and six adult (3 years old) female Tibetan sheep to assess their blood gas indicators, observe lung microstructures, and measure the expression levels of key proteins in the lungs. The results indicated that adult sheep exhibited higher hemoglobin concentrations and finer, denser pulmonary vasculature, which enhanced their oxygen-carrying capacity and increased the surface area available for blood gas exchange, resulting in improved oxygen transfer capacity. Conversely, lambs demonstrated larger lungs relative to their body weight and greater pulmonary vascular volumes, which increased relative pulmonary ventilation and blood flow, thereby enhancing oxygen uptake. These findings suggested that Tibetan sheep employ different adaptation strategies to high-altitude hypoxia at various life stages.

## Introduction

The Tibetan Plateau is the highest plateau on the Earth, with an average altitude exceeding 4,000 m (1), covering approximately a quarter of China’s total territory. Life on the Tibetan Plateau presents myriad environmental challenges, particularly hypoxia due to low barometric pressure. For example, the partial pressure of oxygen (pO_2_) at an altitude of 4,000 m is approximately 60% of that at sea level (2). This harsh environment exerts strong evolutionary pressure on organisms to adapt to high-altitude conditions.

Tibetan sheep arrived on the Tibetan Plateau approximately 3,100 years ago via the Tang-Bo Ancient Road and have since settled permanently (3). After an extended adaptation period, various organs and tissues of Tibetan sheep have undergone significant remodeling, enabling them to thrive in hypobaric hypoxia. Consequently, they have become an ideal model for studying high-altitude hypoxia adaptation. A previous study found that Tibetan sheep exhibited a higher rate of resting respiration than Small-tailed Han sheep (a low-altitude breed) (*p* < 0.05), and the area of mitochondrial cristae membranes (the primary site of aerobic respiration) was greater in various tissues of Tibetan sheep (*p* < 0.05) (4). Moreover, the concentration of hemoglobin (Hb), which serves as the primary carrier and deliverer of oxygen, was found to be higher in Tibetan sheep than in Large-tailed Han sheep (another low-altitude breed) (*p* < 0.05) (5). Tibetan sheep at higher altitudes also exhibited a greater number and area of microvessels in the heart and lungs than their lower-altitude counterparts (6, 7). In addition to the physiological changes mentioned above, several key genes associated with hypoxia adaptation have undergone variations in Tibetan sheep, particularly those in the hypoxia-inducible factor family (8, 9). These physiological and genetic changes in Tibetan sheep yield two main consequences: increased oxygen transfer efficiency and utilization in hypoxic environments, with age playing a significant role in these processes. Wang et al. (10) found that aging can weaken aerobic respiration in the hearts of Tibetan sheep. However, the effects of age on the lungs—one of the most critical organs in adapting to high-altitude hypoxia—remain unclear.

To elucidate the morphological and molecular adaptation mechanisms of Tibetan sheep lungs at different ages under high-altitude hypoxia, we selected lambs (4 months old) and adults (3 years old) and assessed their blood gas indicators. We observed their lung microstructures using electron and optical microscopes, and the expression levels of key proteins related to high-altitude hypoxia adaptation were detected using Western blot (WB) and immunofluorescence techniques. By integrating physiological and molecular data from the blood and lungs, this study found that Tibetan sheep of different ages employ distinct strategies to overcome high-altitude hypoxia.

## Materials and methods

### Sample collection and blood gas indicator measurement

In this study, 12 healthy Tibetan sheep were selected from Haiyan County, Qinghai Province, China, at an altitude of approximately 3,500 m. The group consisted of six 4-month-old female lambs and six 3-year-old adult ewes. In the region where the sampling sites were located, Tibetan sheep are generally weaned before they reach 4 months of age. During the late lactation period, the lambs lived naturally, grazing with their ewes without supplementary feeding. Therefore, the dietary structure and life activities of the 4-month-old lambs were similar to those of the 3-year-old adult sheep, which justified the selection of 4-month-old lambs for this study. Jugular venous blood was collected from all sheep using 5-mL sodium heparin tubes. Pre-slaughter live weight and heart and lung weights were recorded. Parenchymal tissue from the middle of the right lung lobus diaphragmaticus was collected and divided into several portions: One portion was placed in liquid nitrogen for WB analysis, another was placed in 4% paraformaldehyde for immunofluorescence, hematoxylin–eosin (HE), and Weigert resorcinol magenta (WRM) staining analyses, and a third portion was placed in 3% glutaraldehyde for ultrastructural observation using transmission electron microscopy (TEM). The left lung was collected to create a pulmonary artery corrosion cast for structural observation using scanning electron microscopy (SEM).

The collected venous blood was analyzed for blood gas indicators using an i-STAT analyzer (Abbott, Chicago, IL, United States). The measurements included hematocrit (Hct), Hb concentration, partial pO_2_, partial pressure of carbon dioxide (pCO_2_), oxygen saturation (sO_2_), total carbon dioxide, glucose (Glu) concentration, and hydrogen ion concentration (pH). The pH, pO_2_, and sO_2_ were used to calculate the half-saturation oxygen partial pressure (p50) according to the formula by Lichtman et al. (11). The p50 represents the pO_2_ at which 50% of the Hb is bound to oxygen, indicating the affinity of Hb for oxygen.

### Histological staining and morphological observation of the lungs

HE and WRM staining were performed on lung tissue fixed in 4% paraformaldehyde. HE staining dyes the nucleus purple–blue and the cytoplasm and extracellular matrix red, while WRM staining highlights elastic fibers in dark blue. Three fixed lung tissues were selected from both the lamb and adult groups. The samples were dehydrated in ethanol, rinsed in xylene, and then embedded in paraffin. The paraffin-embedded blocks were cut into ultrathin sections using a Leica-2016 microtome (Leica, Wetzlar, Germany) and subsequently stained with HE. Three microscopic fields of view at 100× and 200× magnification were captured for each HE-stained section using a BA210 digital microscope camera (Motic, Xiamen, China) to observe the number and area of arterioles and alveoli, respectively. One paraffin section from each age group was selected for WRM staining, and three different 400× microscopic fields of view were taken to observe the characteristics of the elastic fibers. The HE and WRM staining were conducted by Lilai Biotech Co., Ltd. (Chengdu, China).

For ultrastructural observation of the alveolar septum, one lung tissue sample fixed in 3% glutaraldehyde was selected from each age group. The selected samples were refixed in 1% osmium tetroxide, dehydrated in acetone, and then soaked in a mixture of epoxy resin and acetone. The soaked samples were embedded in epoxy resin, dried, and sectioned into ultrathin sections using an EM-UC7 microtome (Leica, Wetzlar, Germany). The ultrathin sections were stained with uranyl acetate and lead citrate before the ultrastructure of the alveolar septum was observed using a JEM-1400PLUS TEM (Jeol, Tokyo, Japan).

### Corrosion cast making of pulmonary artery and structural observation

To study the branching and distribution characteristics of the pulmonary arteries in lamb and adult Tibetan sheep, corrosion casts of the pulmonary arteries were created and observed using SEM. Casting agents with 10 and 15% concentrations were prepared using acrylonitrile–butadiene–styrene terpolymer pellets mixed with a 1:1 acetone–butanone solution. The left lungs of six sheep from each age group were selected, and casting agents at 10 and 15% concentrations were successively injected from the aorta until the pulmonary arteries were filled. Once the casting agent had completely solidified, the left lung was placed in hydrochloric acid until the lung tissue was completely corroded, forming pulmonary artery corrosion casts. The casts were then cleaned using ultrasound, coated with gold using an E-1045 ion coater (Hitachi, Tokyo, Japan), and subsequently observed with an S-3400 N SEM (Hitachi, Tokyo, Japan).

### WB and immunofluorescence analyses

*Hb beta* (*HBB*) is one of the most important genes in animals’ adaptation to hypoxic environments. In this study, the expression levels of HBB protein in the lung tissue of Tibetan sheep were analyzed using WB and immunofluorescence techniques. Total proteins were extracted from the lung tissues of three Tibetan sheep from each age group and separated by 12% sodium dodecyl-polyacrylamide gel electrophoresis (SDS-PAGE). These proteins were transferred onto polyvinylidene fluoride membranes, blocked, and then incubated with rabbit anti-HBB polyclonal antibody (Abmart, Shanghai, China) and rabbit anti-*β*-tubulin polyclonal antibody (internal reference, Abmart, Shanghai, China) at 4°C overnight. The membranes were incubated with a secondary antibody (goat anti-rabbit immunoglobulin G [IgG], Abmart, Shanghai, China) at room temperature for 2 h. Finally, the blot images were visualized using NcmECL Ultra reagents (NCM Biotech, Suzhou, China) and quantified using AlphaEase FC software.

One paraffin section from each age group was selected for immunofluorescence staining. The sections were blocked with 3% bovine serum albumin (Servicebio, Wuhan, China) and then incubated with rabbit anti-HBB polyclonal antibody (Abmart, Shanghai, China) at 4°C overnight. Subsequently, the sections were incubated with a secondary antibody (goat anti-rabbit IgG, Abmart, Shanghai, China) labeled with fluorescein isothiocyanate at room temperature for 50 min and counterstained with DAPI. The sections were then imaged using CaseViewer software.

### Measurements and statistical analyses

The area and number of arterioles and alveoli in each micrograph field of view of HE-stained sections were measured and counted using Image-Pro Plus 6.0. Three left pulmonary artery corrosion casts were selected from each age group to measure the diameters of the aorta and its first-, second-, third-, and fourth-level branches using digital vernier calipers.

Independent sample *t*-tests were performed using Statistical Package for Social Sciences 19.0 on the blood gas indicators, the proportion of lung and heart weight to body weight, the area and number of arterioles and alveoli, and the proportions of the aorta and first-, second-, third-, and fourth-level branch diameters to lung weight (mm/g, representing vascular volume), as well as the WB quantification results between the two age groups. All experimental data were expressed as mean ± standard deviation, with statistical significance at a *p*-value of <0.05.

## Results

### Differences in blood gas indicators

**Figure 1:**
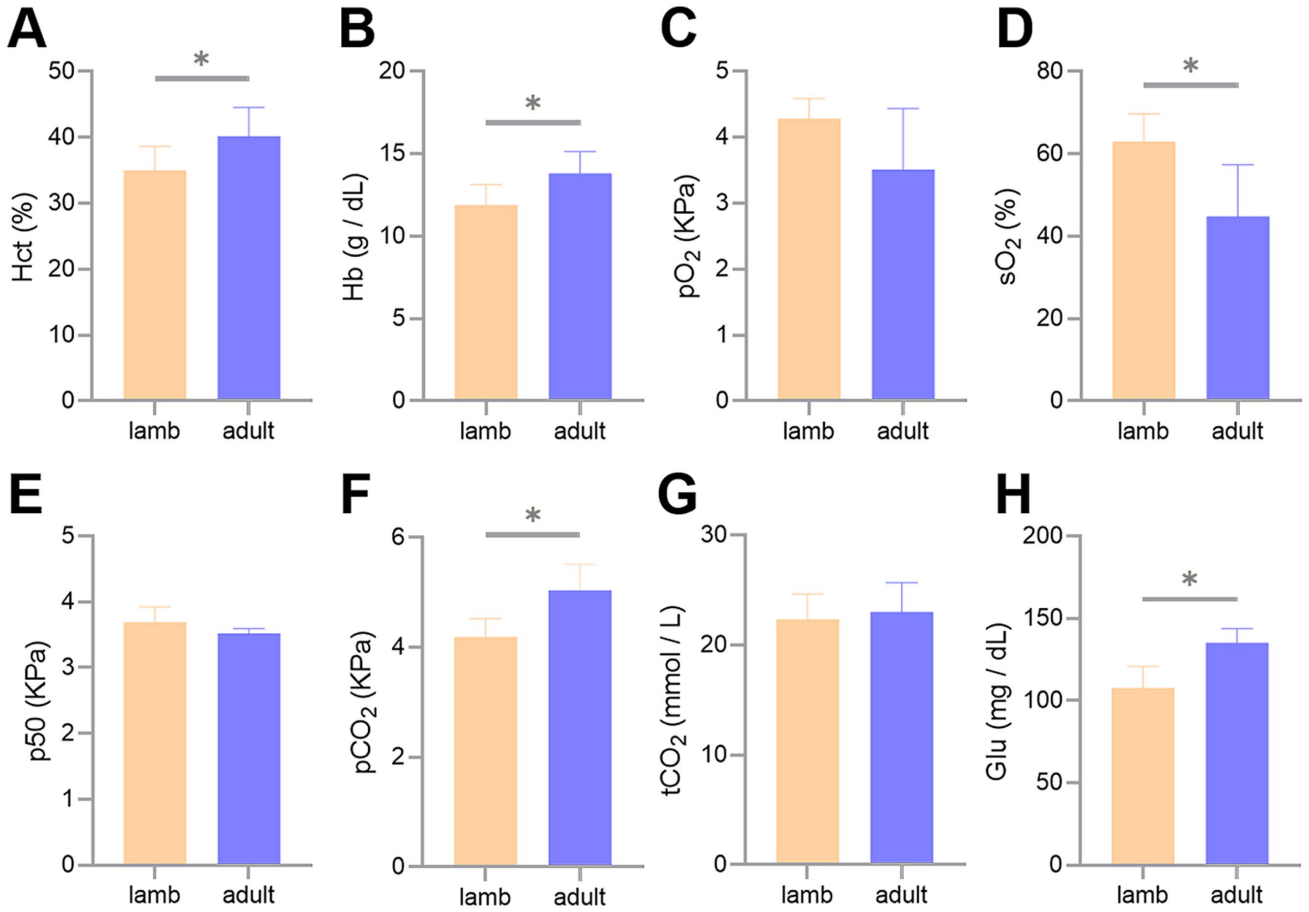
Differences in blood gas indicators between lambs and adult Tibetan sheep. Hematocrit **(A)**, hemoglobin concentrations **(B)**, partial pressure of oxygen **(C)**, oxygen saturation **(D)**, half-saturation oxygen partial pressure **(E)**, partial pressure of carbon dioxide **(F)**, total carbon dioxide **(G)**, and glucose concentrations **(H)** are shown. An asterisk (*) indicates significant differences between different age groups (*p* < 0.05).

The measurement results of the blood gas indicators revealed that Hct, Hb, partial pCO_2_, and Glu concentrations were significantly higher in adult Tibetan sheep than in lambs (*p* < 0.05). In contrast, sO_2_ was lower in adults than in lambs (*p* < 0.05) (Figure 1).

### Morphological differences in the lungs

**Figure 2:**
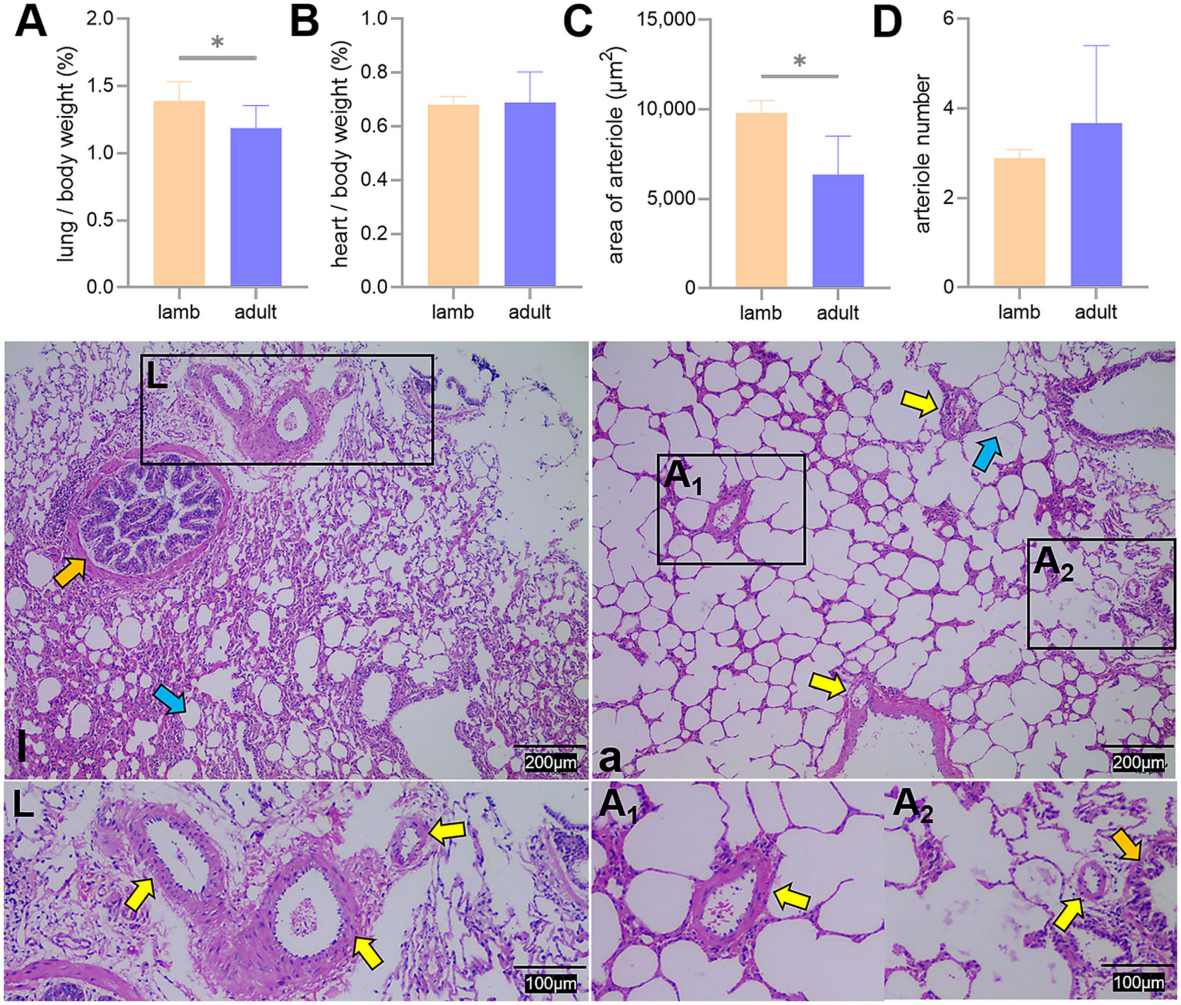
Proportion of lung and heart to body weight, along with lung hematoxylin and eosin staining. Differences in the lung **(A)** and heart **(B)** to body weight ratio, average area **(C)**, and number **(D)** of pulmonary arterioles between lambs (l) and adult (a) Tibetan sheep are presented. Pulmonary arterioles are shown under an optical microscope (l, a). An asterisk (*) indicates significant differences between different age groups (*p* < 0.05). Yellow arrows indicate pulmonary arterioles, orange arrows indicate terminal bronchioles, and blue arrows indicate alveoli.

The ratio of lung weight to body weight and the pulmonary arteriolar area in adult Tibetan sheep were significantly lower than those in lambs (*p* < 0.05). In contrast, the heart-to-body weight ratio and the number of pulmonary arterioles did not differ significantly between the two groups (*p* > 0.05) (Figure 2).

**Figure 3:**
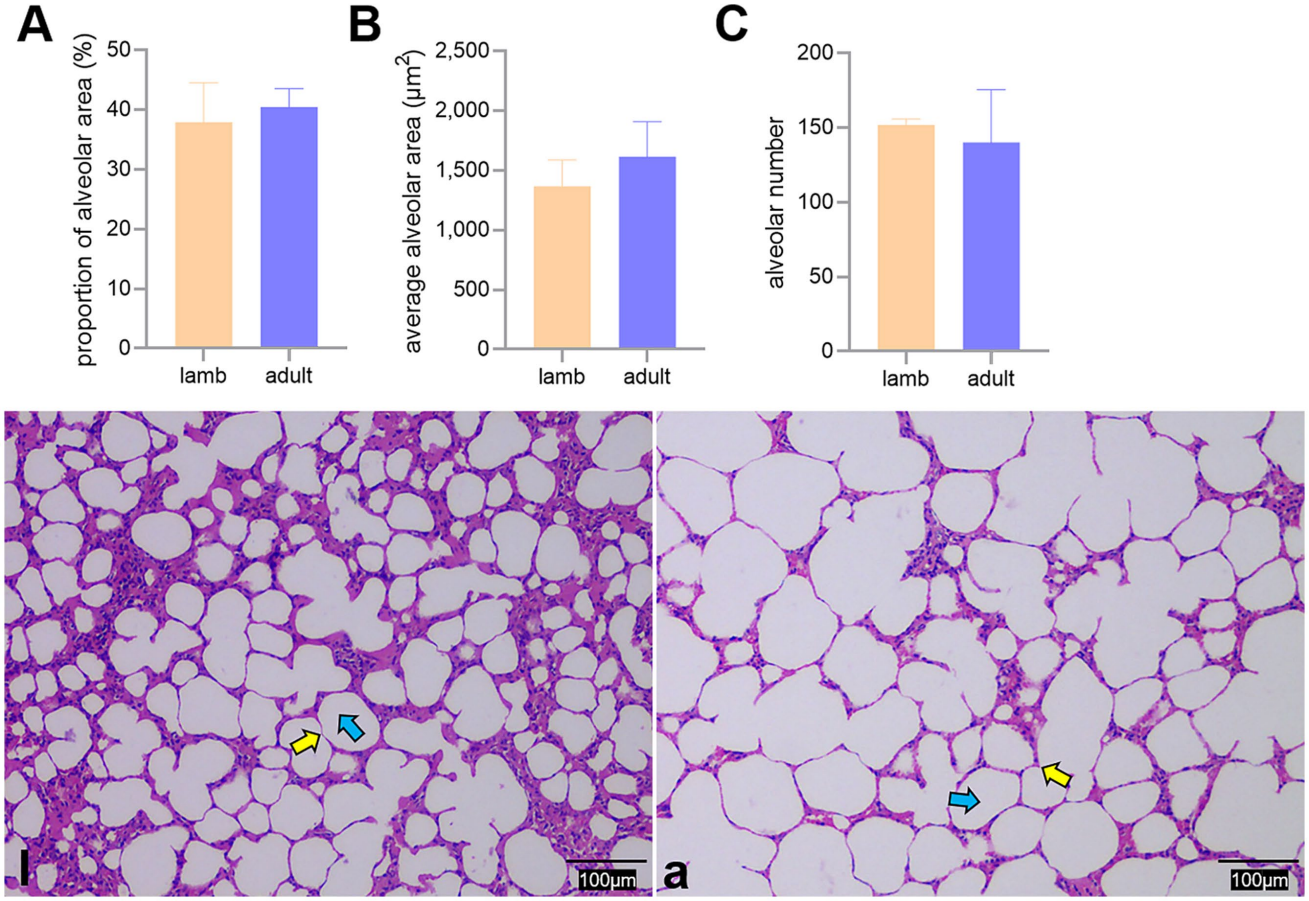
Differences in the proportion of alveoli to the field of view area **(A)**, average area **(B)**, and number **(C)** of alveoli between lambs (l) and adult (a) Tibetan sheep, along with alveoli observed under an optical microscope (l, a). Yellow and blue arrows indicate the alveolar septum and alveoli, respectively.

The proportion of alveoli to the field of view area, the average area, and the number of alveoli did not differ significantly between the two groups (*p* > 0.05) (Figure 3).

**Figure 4:**
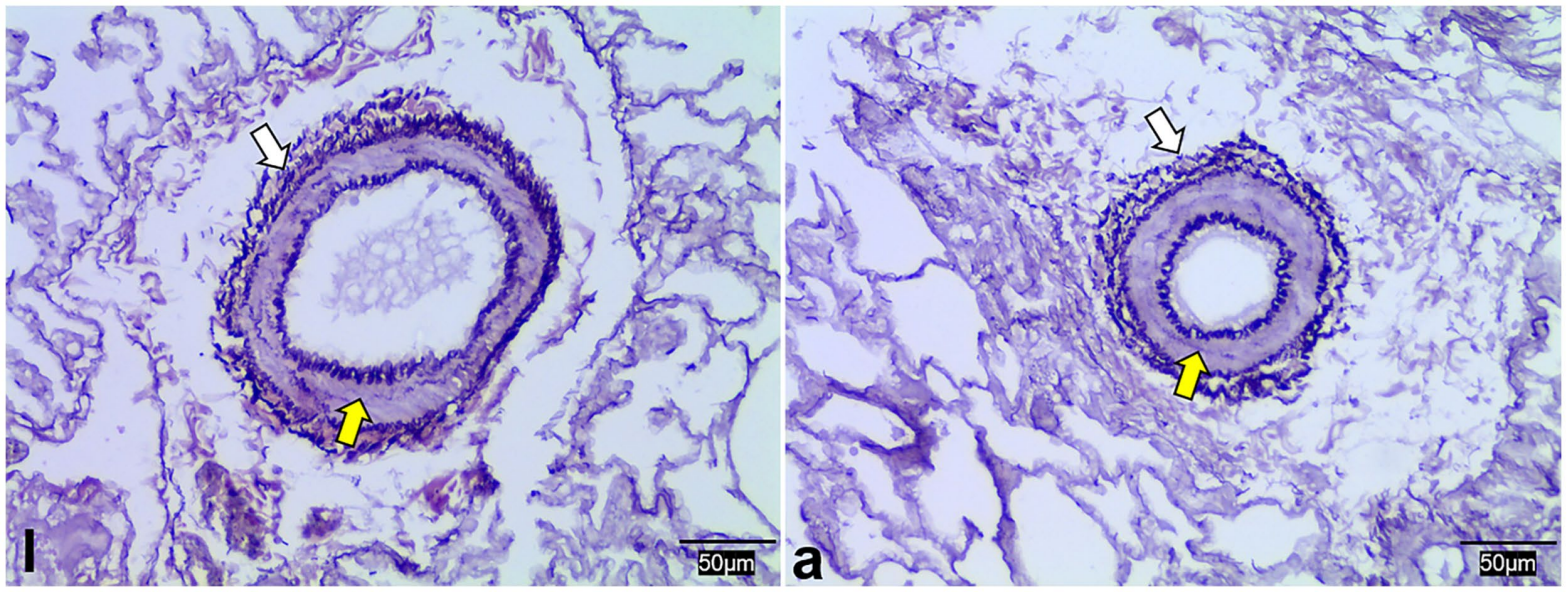
Weigert resorcinol magenta staining of lung elastic fibers in lambs (l) and adult (a) Tibetan sheep. Yellow and white arrows indicate arterioles and elastic fibers, respectively.

WRM staining results demonstrated that the pulmonary arterioles of both lambs and adult Tibetan sheep contained numerous elastic fibers and exhibited thick vascular walls (Figure 4).

**Figure 5:**
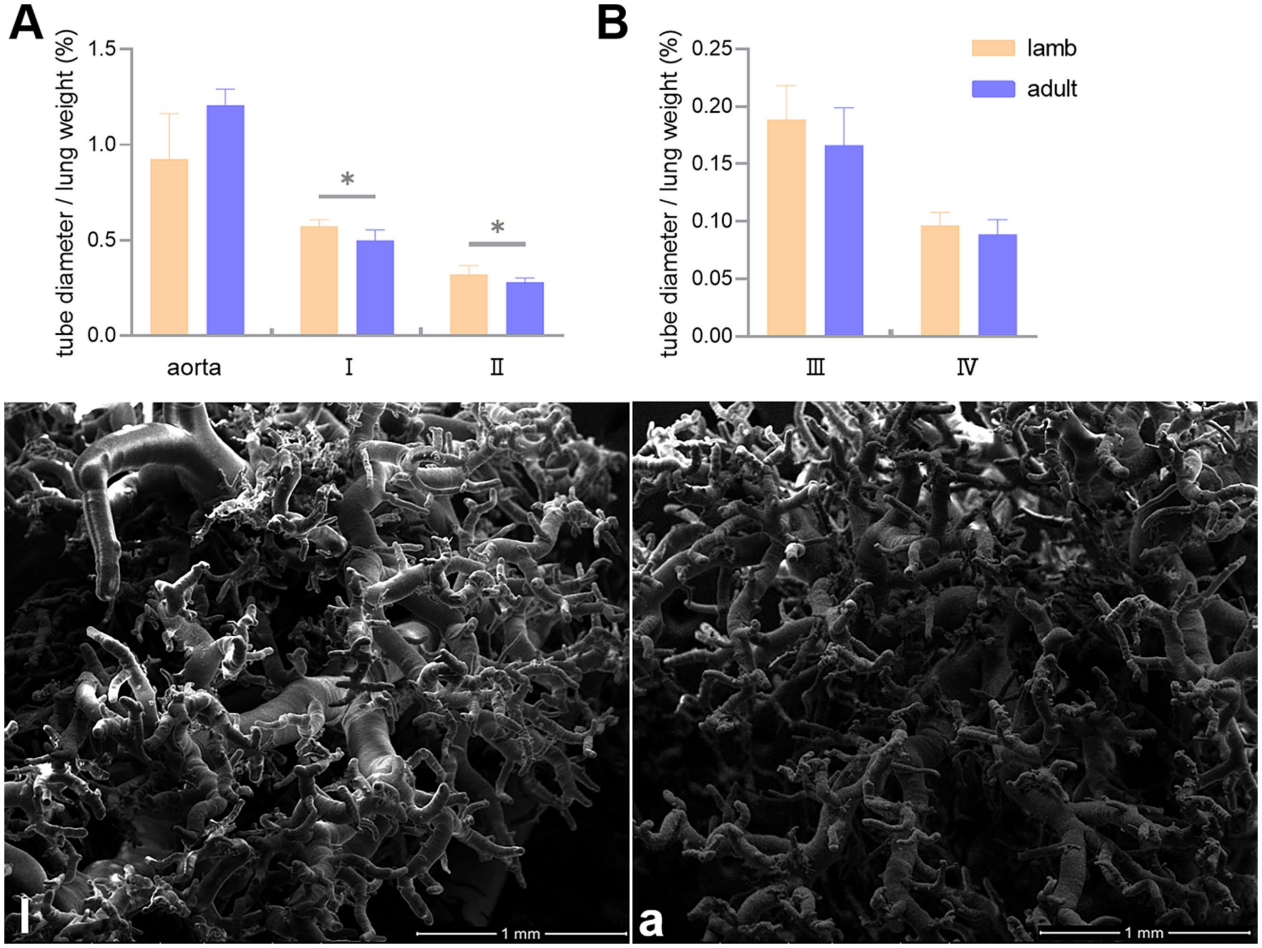
Differences in the ratio of the aorta, first (I), second (II), third (III), and fourth (IV) branch diameters to lung weight (mm/g) between lambs (l) and adult (a) Tibetan sheep **(A,B)**, along with the characteristics of pulmonary artery branches (l,a). An asterisk (*) indicates significant differences between different age groups (*p* < 0.05).

In this study, the ratio of pulmonary artery diameter to lung weight (mm/g) was used to represent vascular volume. It was found that the volumes of the first and second branches of the pulmonary artery were significantly smaller in adult Tibetan sheep than in lambs, except for the aorta and the third and fourth branches (Figures 5A,B). SEM examined the corrosion casts, revealing that adult Tibetan sheep had more branches and thinner diameters in their pulmonary arteries than lambs (Figures 5l,a).

**Figure 6:**
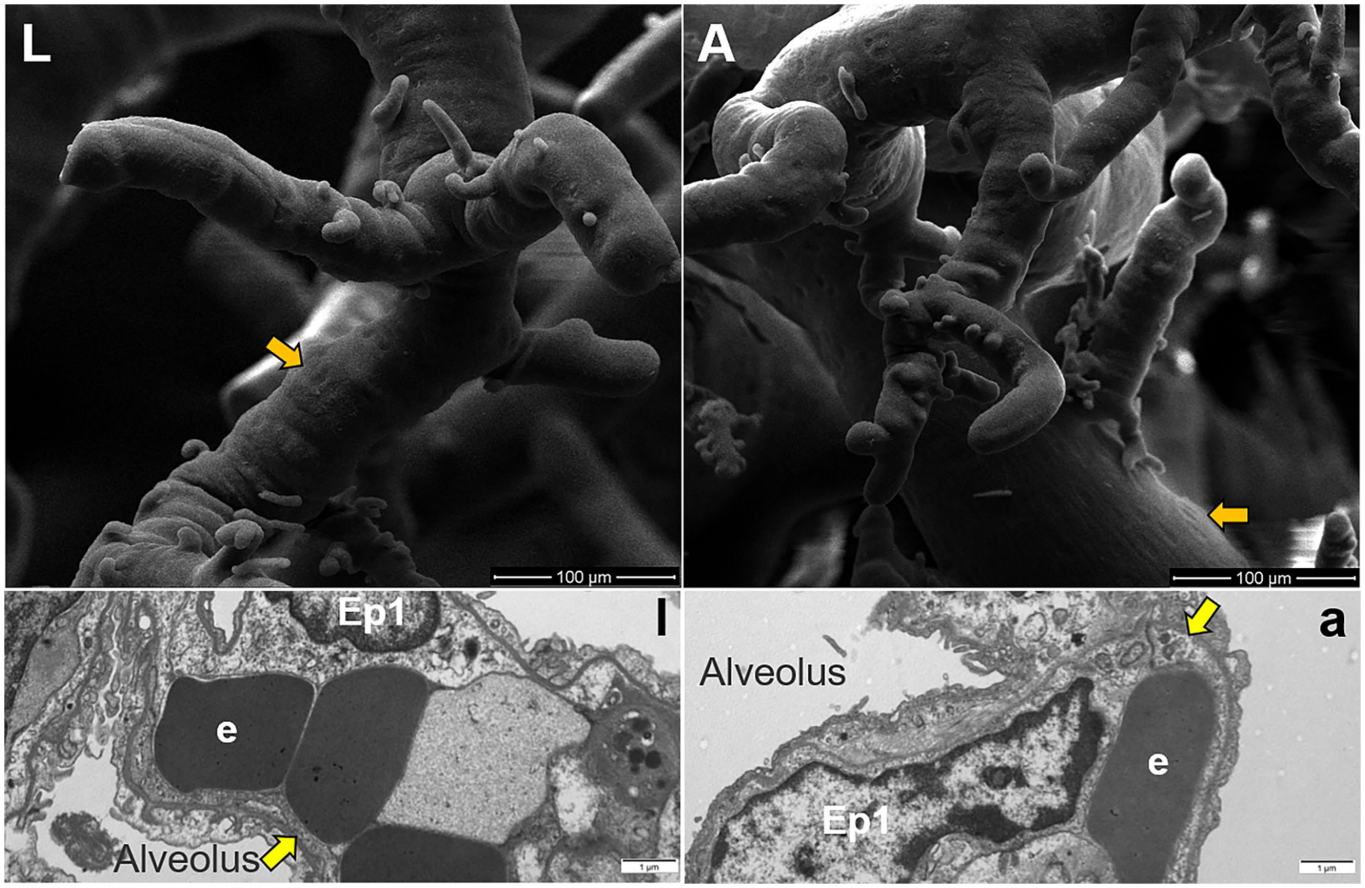
Surface features of pulmonary artery corrosion casts (L,A). Ultrastructure of capillary artery walls in the alveolar septum (l,a), along with type I epithelial cells (Ep1) and erythrocytes (e). Yellow arrows indicate vascular endothelial cells, whereas orange arrows indicate endothelial cell indentations.

The surface characteristics of the fine branches of pulmonary artery corrosion casts were observed using SEM, which revealed that adult Tibetan sheep exhibited deeper and denser indentations of endothelial cells than lambs (Figures 6L,A). TEM was used to examine the alveolar septum, showing that the vascular endothelial cells in adult Tibetan sheep were larger and exhibited muscularization than those in lambs (Figures 6l,a).

### Differences in expression of HBB in the lungs

**Figure 7:**
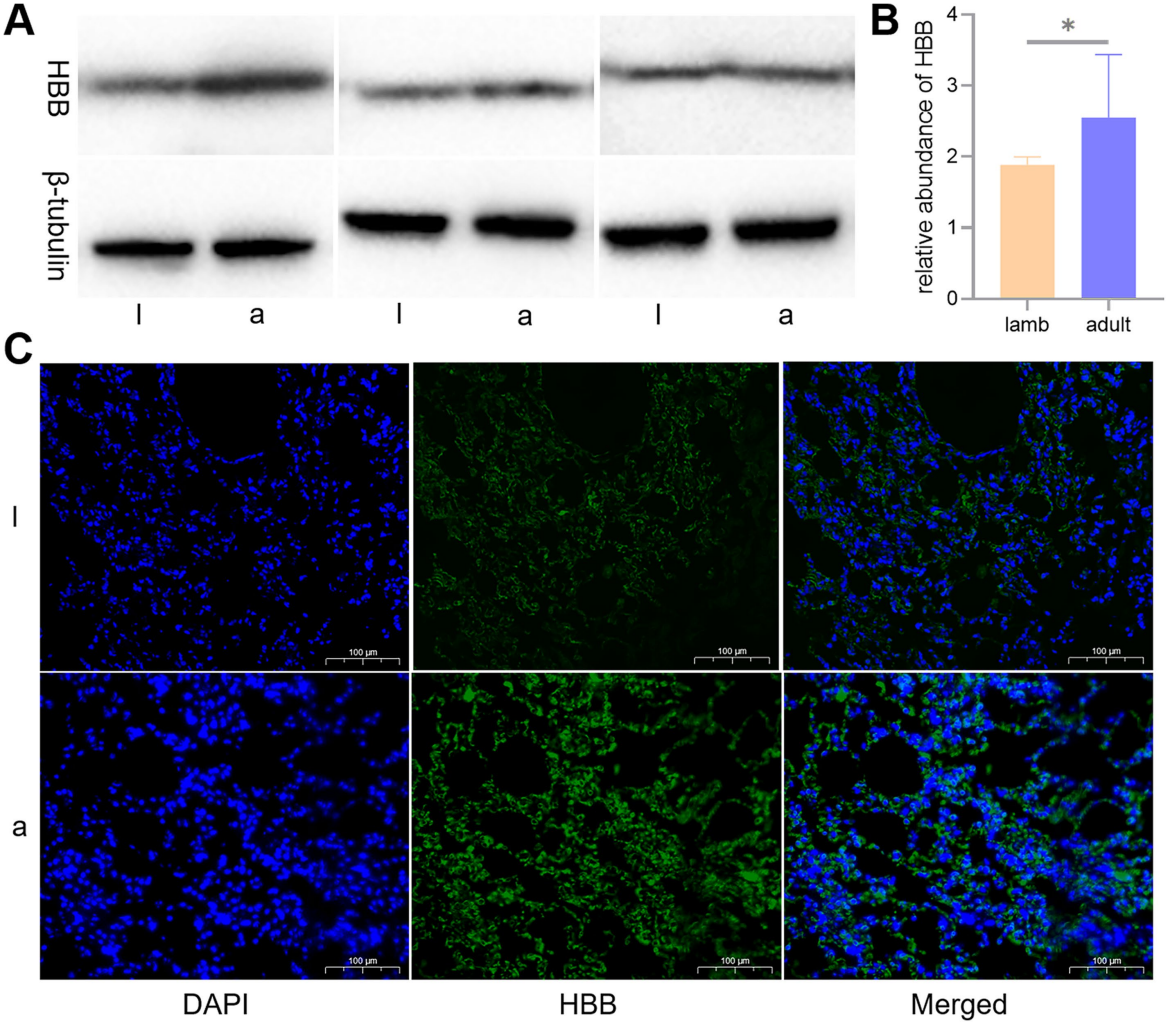
Western blot (WB) and immunofluorescence analyses of the key protein, hemoglobin beta (HBB). Differences in the expression levels of HBB protein in lambs (l) and adult (a) Tibetan sheep are depicted by WB **(A)**, a histogram of WB test results **(B)**, and immunofluorescence **(C)**.

To determine the expression levels of HBB protein, we conducted WB and immunofluorescence analyses. The WB results indicated a significant difference in the expression of HBB protein in the lungs of lambs and adult sheep (*p* < 0.05). The immunofluorescence tests corroborated the results obtained from the WB analysis (Figure 7).

## Discussion

The blood gas indicators of Tibetan sheep play a crucial role in overcoming high-altitude hypoxia, and age may influence these indicators. In this study, we found that adult Tibetan sheep exhibited higher Hct, Hb, pCO_2_, and Glu concentrations, along with sO_2_, than lambs (*p* < 0.05) (Figure 1). In bovid artiodactyls, the prenatal expression of fetal Hb isoforms with high blood oxygen affinity helps maintain appropriate oxygen concentrations in the uterus (12). The activity of this Hb isoform may persist postnatally (13). Sheep possess a gene block in the *β*-globin gene cluster, including a specialized β-globin paralog (*β^C^*) (14, 15). During the juvenile stage, the *β^C^* gene is highly expressed. It contributes to the formation of the juvenile Hb isoform (HbC) (16), which has a higher blood oxygen affinity than the adult Hb isoform (17). Consequently, lambs, with Hb isoforms with a high blood oxygen affinity, can afford a moderate reduction in Hct and Hb concentrations to decrease blood viscosity, thereby alleviating pressure on the developing heart and vascular system while increasing sO_2_.

Consistent with this, WB and immunofluorescence analyses indicated an elevation in HBB expression in the lungs of adult Tibetan sheep (*p* < 0.05) (Figure 7). It was noted that the *HBB* gene was inherited from *Argali* (*Ovis ammon*) and positively selected during the dispersal of Tibetan sheep across the Tibetan Plateau (3). Lefrancais et al. (18) identified the lungs as an organ with significant hematopoietic potential, while Patel et al. (19) demonstrated that the arterial endothelium can produce hematopoietic stem cells. The presence of HBB protein in the lungs of Tibetan sheep may arise from the lungs’ own hematopoiesis and erythrocytes trapped in the lung’s capillaries. Given that circulating blood frequently exchanges with reserve blood, it can be inferred that Hb in the lung is equivalent to Hb in the erythrocytes. HBB proteins are essential subunits of Hb; thus, the expression of HBB protein in the lungs further validates the accuracy of the blood Hb concentration measurements. Similarly, Hb concentrations were higher in Tibetan sheep than in Large-tailed Han sheep raised at lower altitudes (5). As the most significant biomacromolecule for maintaining blood acid–base balance, Hb primarily facilitates the transport of oxygen and carbon dioxide (20). An increase in Hb concentration leads to a corresponding rise in the total amount of oxygen absorbed, subsequently elevating pCO_2_. Therefore, pCO_2_ exhibits a trend parallel to Hb concentration, consistent with the blood gas indicator data from this study. Moreover, carbohydrates provide more energy than fatty acids per unit of oxygen consumed; thus, the energy supply of high-altitude indigenous species shifts toward Glu oxidation and glycolysis for efficient utilization of scarce oxygen (21, 22). Furthermore, the enzymes responsible for transporting and oxidizing fatty acids are not fully developed in juvenile animals (23, 24), making this shift in energy supply more pronounced in juvenile animals and resulting in lower plasma Glu levels, similar to the findings in humans (25). Consistent with this, Glu concentrations were lower in lambs than in adult Tibetan sheep in this study. Collectively, the blood gas indicators of Tibetan sheep exhibit age-related changes as they adapt to chronic hypoxia.

In addition to blood gas indicators, lung morphology in Tibetan sheep varies by age. In this study, the lung-to-body weight ratio of lambs was found to be greater than that of adult Tibetan sheep (*p* < 0.05), whereas the heart-to-body weight ratio showed no significant difference (*p* > 0.05) (Figures 2A,B). It is well established that in the early stages of life, both in humans and animals (e.g., fetal and juvenile stages), the internal organs and brain develop preferentially. Consequently, the proportion of internal organs to body weight is higher in juveniles than in adults. This phenomenon may reflect the “principle of priority development,” which occurs when organisms develop under nutritional limitations. Moreover, morphological differences in the lungs of Tibetan sheep across different ages are primarily evident in the alveoli and pulmonary vessels. This study revealed that the alveoli-to-field-of-view area ratio and average alveolar area in adult Tibetan sheep were larger than those in lambs. However, the total number of alveoli was smaller (*p* > 0.05) (Figure 3). Human studies have demonstrated a significant increase in alveolar area with age (26–28). While the difference in alveolar area between lambs and adult Tibetan sheep was not statistically significant, there was a noticeable trend toward increased alveolar area in adults. The discrepancy between our findings and those observed in humans may be attributed to the limited sample size of this study. In contrast to lambs, adult Tibetan sheep engage in more life activities, such as breeding and feeding their young. An increased alveolar area enhances the surface area available for blood gas exchange; however, adequate oxygen supply for these activities also necessitates a well-developed pulmonary vascular network to facilitate oxygen diffusion into the bloodstream, which is then transported throughout the body via left ventricular contraction.

Regarding pulmonary vessels, this study found that adult Tibetan sheep had a significantly lower (*p* < 0.05) pulmonary arteriolar area (Figure 2C) and a reduced diameter of the first and second pulmonary artery branches relative to lung weight, indicating a smaller pulmonary vascular volume (Figure 5A). Furthermore, the pulmonary arteries of adult Tibetan sheep exhibited more branches and thinner diameters (Figures 5l,a). This finer and denser pulmonary vasculature, coupled with the larger alveolar area, creates more sites for blood gas exchange, suggesting that lung development in adult Tibetan sheep is more advanced. Similarly, in humans, both the alveoli and pulmonary vessels have been shown to increase in size with age (29). In addition, the walls of the pulmonary arterioles in adult Tibetan sheep may be thicker than those in lambs (Figure 4), which is consistent with previous findings indicating that chronic hypoxia can lead to thickening of the pulmonary artery (30–32). A thickened pulmonary artery contains more smooth muscle, enhancing its ability to undergo vasodilation and vasoconstriction, which is crucial for avoiding pulmonary hypertension, a common affliction in high-altitude environments. SEM was used to examine the surface characteristics of pulmonary artery corrosion casts, revealing deeper and denser indentations of endothelial cells in adult Tibetan sheep (Figures 6L,A). This suggests that the thickened pulmonary artery has improved contractile ability, facilitating better blood flow control. TEM observations of the alveolar septum indicated that the endothelial cells of the capillary artery walls in adult Tibetan sheep displayed muscularization (Figures 6l,a), further reflecting the contractile potential of the pulmonary artery. Conversely, lambs possess certain advantages; specifically, their larger lungs relative to body weight facilitate high relative pulmonary ventilation, and their larger pulmonary vascular volumes support increased blood flow, ultimately enhancing oxygen uptake capacity.

## Conclusion

Tibetan sheep of different ages exhibit distinct adaptation strategies to high-altitude hypoxia. Adult sheep address hypoxia by increasing Hb concentration and enhancing the surface area for blood gas exchange, thereby improving oxygen transfer capacity. In contrast, lambs adapt to hypoxia by increasing relative pulmonary ventilation and blood flow, which enhances their oxygen uptake ability. In addition, lambs may rely on Hb isoforms with a higher affinity for oxygen, further increasing their oxygen-binding capacity. These findings provide valuable insights into the survival mechanisms of Tibetan sheep and other indigenous species on the Tibetan Plateau.

## Data Availability

The original contributions presented in the study are included in the article, further inquiries can be directed to the corresponding author.
